# Self-reported pain scores as a predictor of preterm birth in symptomatic twin pregnancy: a retrospective study

**DOI:** 10.1186/s12884-021-03931-1

**Published:** 2021-07-01

**Authors:** Ji Hoi Kim, Seung Mi Lee, Sungyoung Lee, So Yeon Kim, Hye Jeong Hue, Chan-Wook Park, Joong Shin Park, Jong Kwan Jun

**Affiliations:** 1 Department of Obstetrics and Gynecology, Seoul National University College of Medicine, Seoul, South Korea; 2 Center for Precision Medicine, Seoul National University Hospital, Seoul, South Korea; 3 Institute of Reproductive Medicine and Population, Medical Research Center, Seoul National University College of Medicine, Seoul, South Korea

**Keywords:** Preterm labor, Preterm birth, Self-reporting pain score, Numerical rating scale pain score, Symptomatic twin pregnancy, Fetal fibronectin, Cervical length

## Abstract

**Background:**

To evaluate the self-reported pain scores as a predictor of preterm birth (PTB) in symptomatic twin pregnancy and to develop a nomogram for the prediction model.

**Methods:**

We conducted a retrospective study of 148 cases of symptomatic twin pregnancies before 34 weeks of gestation visited at Seoul national university hospital from 2013 to 2018. With other clinical factors, self-reported pain score was evaluated by the numerical rating scale (NRS) pain scores for pain intensity. By multivariate analyses and logistic regression, we developed a prediction model for PTB within 7 days. Using the Cox proportional hazards model, the curves were plotted to show the predictability of the PTB according to NRS pain score, while adjusting the other covariates.

**Results:**

Twenty-three patients (15.5 %) delivered preterm within 7 days. By a logistic regression analysis, higher NRS pain score (OR 1.558, 95 % CI 1.093–2.221, *P* < 0.05), shorter cervical length (OR 3.164, 95 % CI 1.262–7.936, *P* < 0.05) and positive fibronectin results (OR 8.799, 95 % CI 1.101–70.330, *P* < 0.05) affect PTB within 7 days. Using the variables, the area under the receiver operating characteristic curve (AUROC) of the prediction model was 0.917. In addition, we developed a nomogram for the prediction of PTB within 7 days.

**Conclusions:**

Self-reported pain scores combined with cervical length and fetal fibronectin are useful in predicting impending PTB in symptomatic twin pregnancy.

## Background

Preterm birth (PTB) is one of the most important problems in twin pregnancy. In spite of developments in the management of preterm labor, such as antenatal corticosteroids or tocolytics, the risk of preterm birth in twin pregnancy is still high, with a reported incidence of 19.8 % for PTB < 34 weeks and 59.9 % for PTB < 37 weeks [1].

Preterm labor is one of the most frequent problems for antenatal admission to the hospital. It made up about one-third in US hospitals [2, 3]. However, patients with threatened preterm labor finally result in preterm birth in only 22 % of cases [4], and accurate prediction of impending preterm birth is an important issue in the clinical decision regarding the administration of antenatal corticosteroid or tocolytics and transfer to referral hospitals.

As good candidates for predictive markers, sonographic cervical length measurement and fetal fibronectin test are well studied in asymptomatic and symptomatic twin pregnancy [5–8]. But the results were controversial and not encouraging.

For the evaluation of pain intensity, self-reported pain scales are the most commonly used method to perform mathematical calculations in a variety of areas, including postoperative pain, orthopedic pain, and obstetric pain [9]. Among several self-reported pain scores, the numerical rating scale (NRS) pain score is preferred, because it is more responsive and sensitive than other pain scores and it is easy to understand by patients [10]. In obstetrics, the NRS pain score has been used as a good indicator of labor pain [11–13]. However, the clinical usefulness of the NRS pain score in preterm labor has not been determined, even though preterm labor itself is associated with labor pain [14].

The purpose of this study was to demonstrate whether self-reported pain scores can predict impending preterm birth in twin pregnancy with preterm labor and to develop a nomogram for the prediction of preterm birth.

## Methods

### Study design and population

It is a single-center retrospective cohort study. The institutional review board of Seoul National University Hospital Clinical Research Institute approved this study (No 1301-129-462, date of approval 2013-02-20) and has waived the requirement of the informed consent for this study. All methods were performed in accordance with the relevant guidelines and regulations.

This study population consisted of consecutive twin pregnancies who visited Seoul National University Hospital with symptomatic preterm uterine contractions from March 2013 to January 2018 and met the following inclusion criteria: (1) gestational age < 34 weeks at the time of visit; (2) without evidences of ruptured membranes; (3) both alive fetuses.

### Data collection

At the time of the visit, physical examinations were performed including digital vaginal examinations for cervical effacement and dilatation. Patients with ruptured membranes were excluded, and rupture of the membrane was diagnosed by speculum examinations with a combination of the presence of amniotic fluid leakage or pooling, positive nitrazine or amnisure test. A swab was taken in the posterior fornix and analyzed for a fetal fibronectin test. Transvaginal ultrasonography was performed for the evaluation of cervical length and funneling.

In our institution, it is a routine practice to obtain clinical information regarding the frequency of uterine contractions and the numerical rating scale (NRS) pain score during uterine contractions in patients with threatened preterm birth. The frequency of uterine contractions was determined based on the cardiotocography. NRS pain score was estimated by patients for pain intensity to be expressed as 0 to 10. These data was collected from the electrical medical records. Patients were classified into two groups according to the interval-to-delivery. Interval-to-delivery was determined as the time interval between the date of the visit for symptomatic uterine contractions and the date of delivery. Clinical factors were compared between women who delivered within 7 days and those who delivered 7 days or later.

### Clinical management

The use of tocolytics therapy or corticosteroid administration was decided at the discretion of the attending physician. Antibiotics are not routinely used in patients with preterm labor, especially without the evidence of chorioamnionitis.

### Statistical analysis

Proportions of categorical variables were compared using the Fisher’s exact test, and Mann-Whitney U test analyzed comparisons of continuous variables. There were described by median with interquartile range for continuous variables and by numbers and percentages for categorical variables. To develop the prediction model for preterm birth within 7 days, multiple logistic regression with backward elimination was performed with clinical variables selected from univariate analysis (*p* < 0.05) according to the interval-to-delivery. We performed 3-fold cross-validation to compute the area under the receiver operating characteristic curves (AUC) of the nomograms for prediction of preterm birth from stepwise selection by AUC using leave-one-out cross validation. As results, the nomogram for prediction of preterm birth within 7 days was built with the variables that remained significant in logistic regression analysis. Additionally, cut-off value of NRS pain score was estimated by the receiver operating characteristic (ROC) curve. Using the Cox proportional hazards model, the curves were plotted to show the predictability of the preterm birth according to NRS pain score, while adjusting the other covariates. A prior sample size calculation was performed to determine how many pregnant women would be needed to detect an increased risk for preterm birth from 10 to 30 % in women with high NRS score. In the literature, the risk of preterm birth was reported as 20 % in twin preterm labor [4]. With 80 % power and a type 1 error of 5 %, we determined that we would require 137 women, with an estimated frequency of 30 % of high NRS (≥ 4) in preterm labor. The data were analyzed with IBM SPSS statistics version 25 software and R statistical computing language. *P* < 0.05 was considered significant.

## Results

Between March 2013 and January 2018, a total of 148 twin pregnancies met the inclusion criteria. Among these 148 women, 23 women (15.5 %) delivered preterm within 7 days. Table 1 compares the clinical characteristics according to interval-to-delivery. Patients who were delivered of their babies at preterm within 7 days had higher NRS pain scores during uterine contractions (median NRS pain score, 4 in patients with interval-to-delivery < 7days vs. 3 in patients with interval-to-delivery ≥ 7days, *p* < 0.001). In addition, patients who were delivered at preterm within 7 days had more frequent uterine contractions, shorter cervical length, a more dilated cervix, and a higher rate of positive fibronectin test.

**Table 1:** Comparison of predictive factors between < 7 days and ≥ 7days of interval-to-delivery

Baseline characteristics	Interval-to-delivery < 7 days (*n* = 23)	Interval-to-delivery ≥ 7 days (*n* = 125)	*p*
Age (yrs)	34.00 (31.00–35.00)	33.00 (31.00–35.00)	NS
Height (cm)	162.60 (157.00-166.40)	162.50 (158.70–166.00)	NS
Weight (kg)	67.00 (59.85–76.65)	63.35 (57.25–70.80)	NS
Body mass index (kg/m2)	27.03 (21.87–29.35)	23.47 (22.29–26.77)	NS
Nulliparity (%)	19/23 (82.6)	94/125 (75.2)	NS
Prior preterm birth (%)	0/23 (0.0)	7/125 (5.6)	NS
Numerical rating scale pain score	4.00 (3.00–6.00)	3.00 (2.00-3.25)	< 0.001
Frequency of uterine contractions (numbers/10mins)	3.00 (2.00–3.00)	2.00 (0.55-3.00)	< 0.05
Cervical length (cm)	0.29 (0.00-0.93) (*n* = 12)	2.01 (1.20–3.01) (*n* = 113)	< 0.001
Cervical dilatation (cm)	2.00 (1.00–3.00)	0.00 (0.00–1.00)	< 0.001
Funneling (%)	9/20 (45.0)	28/113 (24.8)	NS
Positive fetal fibronectin (%)	14/16 (87.5)	36/88 (40.9)	< 0.005
Tocolytics (%)	16/23 (69.6)	53/125 (42.4)	< 0.05
Gestation age at visit(weeks)	31.30 (27.42–32.90)	29.00 (25.51–31.41)	NS
**Delivery outcome**			
Gestation age at delivery(weeks)	31.57 (27.57–33.70)	36.70 (34.27–37.30)	< 0.001
Interval to delivery (days)	1.00 (0.00–4.00)	43.00(30.50–66.50)	< 0.001

Data expressed as median (interquartile range) for continuous variables; n (percentage) for categorical variables

*NS* not significant

**Fig. 1:**
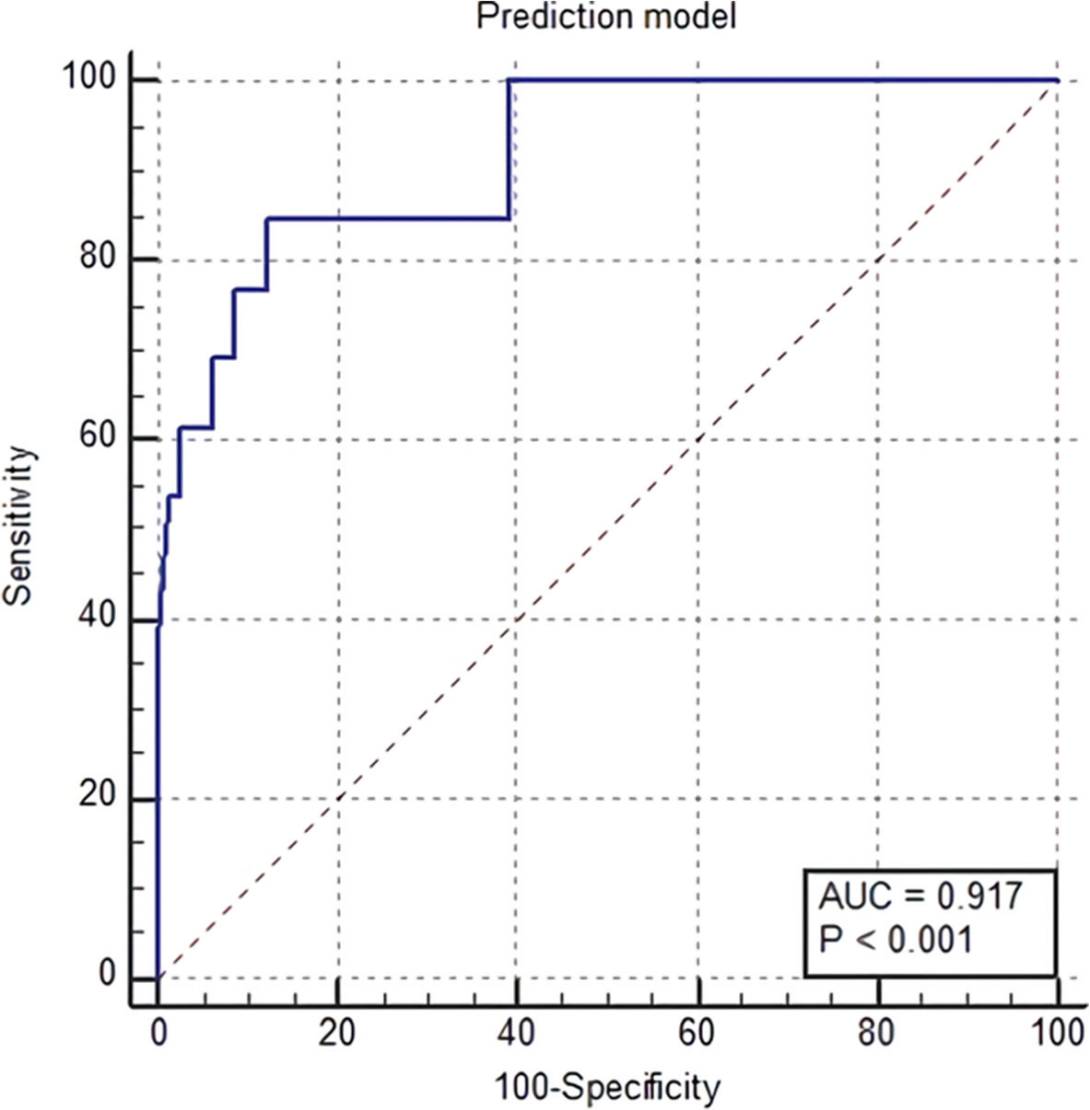
The area under the receiver operating characteristic curve (AUC) of numerical rating scale pain score, cervical length and fetal fibronectin for preterm birth within 7 days

The association between the risk of preterm birth within 7 days and a high NRS pain score remained significant even after adjusting for confounding variables, as noted in Table 2. Higher NRS pain score (Odd ratio (OR) 1.558, 95 % CI 1.093–2.221, *P* < 0.05), shorter cervical length (OR 3.164, 95 % CI 1.262–7.936, *P* < 0.05) and positive fibronectin results (OR 8.799, 95 % CI 1.101–70.330, *P* < 0.05) affect preterm delivery within 7 days in symptomatic twin pregnancies. Using final clinical variables retained in the prediction model of preterm birth within 7 days, the AUC of the prediction model was 0.917 in Fig. 1.

**Table 2:** Logistic regression of significant predictive factors for delivery within 7 days of presentation

	Odds ratio	95 % CI	*p*
Numerical rating scale pain score	1.558	1.093–2.220	< 0.05^*^
Cervical length (cm)	3.174	1.262–7.936	< 0.05^*^
Fibronectin (%)	8.772	1.097–70.135	< 0.05^*^

^*^Adjusted for frequency of uterine contractions, nulliparity, cervical dilation, numerical rating scale pain score, fetal fibronectin, tocolytics, cervical length

**Fig. 2:**
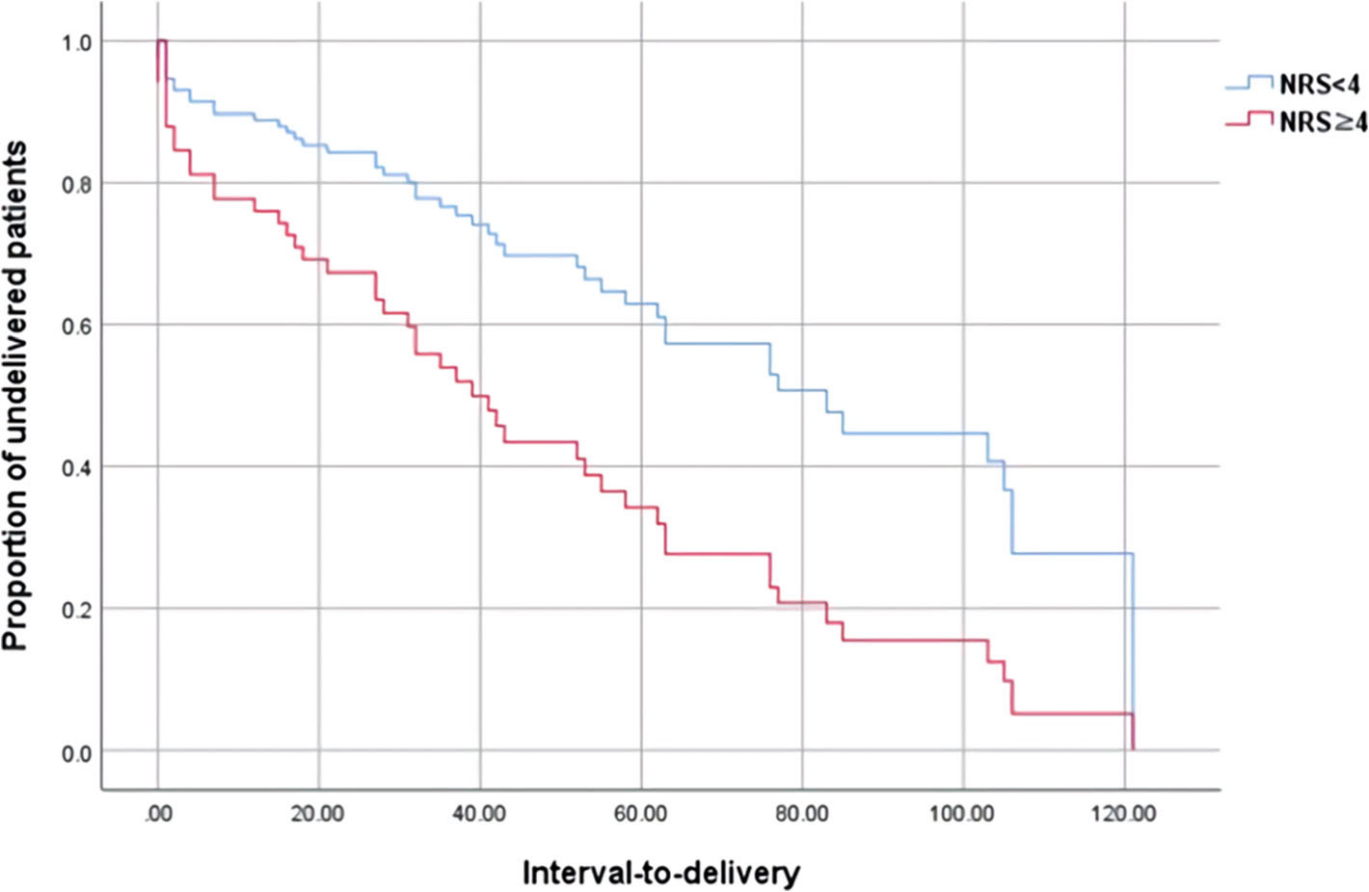
The interval to delivery with numerical rating scale score ≥ 4 after adjusting variables for cervical length and fetal fibronectin

In addition, an NRS pain score was analyzed for estimated cut-off value by ROC curve. The cut-off value for prediction of preterm birth within 7 days was an NRS pain score of 4. Table 3 compares the clinical characteristics by NRS pain score < 4 and ≥ 4. The higher the NSR pain score, the shorter the interval-to-delivery and the higher the risk for preterm birth (< 7 days, < 14 days, < 34 weeks, < 37 weeks). In Fig. 2, the interval-to-delivery was significantly shorter in patients with an NRS pain score ≥ 4 than those with an NRS pain score < 4, even after adjustment for cervical length and fibronectin result (OR 8.03, 95 % CI 1.69–38.24, *p* < 0.01).

**Table 3:** Comparison of clinical factors between numeric rating scale pain score < 4 and ≥ 4 in preterm labor at < 34 weeks of gestations

	Numeric rating scale pain score < 4 (*n* = 103)	Numeric rating scale pain score ≥ 4 (*n* = 45)	*p*
Age (year old)	33.00 (30.00–35.00)	33.00 (31.00–35.00)	NS
Height (cm)	163.00(159.20-166.40)	161.00(157.00-165.60)	NS
Weight (kg)	64.60 (58.00–72.00)	3.90 (56.60-70.55)	NS
Body mass index (kg/m2)	23.57 (22.29–27.25)	24.73 (22.08–26.84)	NS
Nulliparity (%)	85/103 (82.5)	28/45 (62.2)	< 0.05
Prior preterm birth (%)	4/103 (3.9)	3/45 (6.7)	NS
Frequency of uterine contractions (numbers/10mins)	2.00 (0.60-3.00)	3.00 (1.00-3.75)	< 0.05
Cervical length (cm)	1.98 (1.20–2.91)	0.98 (0.00-2.64)	< 0.01
Cervical dilatation (cm)	0.00 (0.00–1.00)	1.00 (0.00–2.00)	< 0.001
Funneling (%)	23/95 (24.2)	14/38 (36.8)	NS
Positive fetal fibronectin (%)	32/73 (43.8)	18/31 (58.1)	NS
Tocolytics (%)	42/103 (40.8)	27/45 (60.0)	< 0.05
Gestation age at visit(weeks)	28.90 (26.00-31.42)	29.85 (25.55–32.20)	NS
**Delivery outcome**			
Gestational age at delivery (weeks)	36.60(34.00-37.30)	34.10 (29.62–37.15)	< 0.01
Interval to delivery (days)	43.00 (28.00–63.00)	20.00 (1.00–46.00)	< 0.01
Delivery at ≤ 34 wks (%)	27/103 (26.2)	22/45 (48.9)	< 0.01
Delivery at ≤ 37 wks (%)	57/103 (55.3)	33/45 (73.3)	< 0.05
< 7 days for interval-to-delivery (%)	8/103 (7.8)	15/45 (33.3)	< 0.05^*^
< 14 days for interval-to-delivery (%)	13/103 (12.6)	20/45 (44.4)	< 0.001

Data expressed as median (interquartile range) for continuous variables; n (percentage) for categorical variables

*NS* not significant

^*^significant after adjustment for nulliparity, frequency of uterine contractions, cervical length, cervical dilation, positive fetal fibronectin

**Fig. 3:**
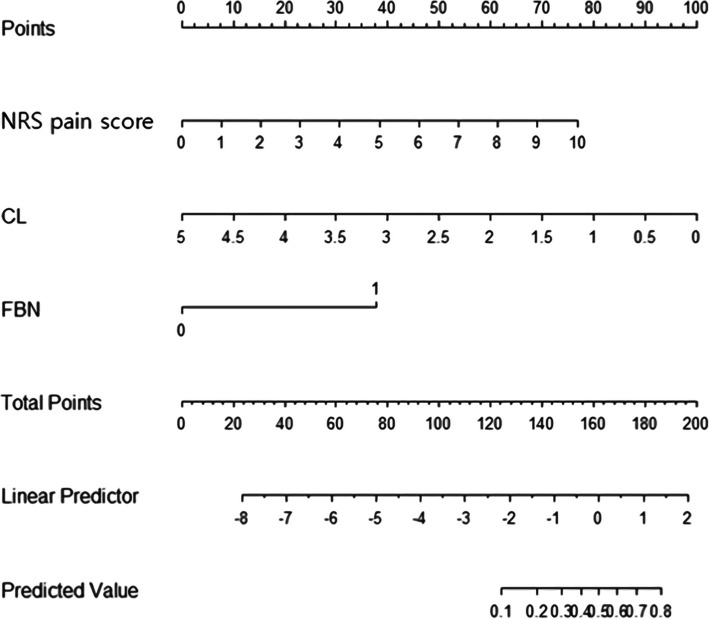
Nomogram for prediction of preterm birth within 7 days with symptomatic uterine contraction in twin pregnancies. NRS pain score, numerical rating scale pain score (0 ~ 10); CL, cervical length (cm); FBN, fetal fibronectin (0: negative, 1: positive)

From the above results, we developed a static nomogram for the prediction of preterm birth within 7 days in Fig. 3. After developing a nomogram, we posted them online (http://lsy.io/nomogram/painscore) for easy access to promote clinical use.

## Discussion

In the present study, patients who delivered within 7 days had a higher NRS pain score during uterine contractions, more frequent uterine contractions, shorter cervical length and a higher rate of positive fibronectin test. In logistic regression analysis, NRS pain score, cervical length, and positive fibronectin were significant risk factors for preterm birth within 7 days. And by using three factors, we developed a static nomogram for prediction of preterm birth within 7 days.

Many papers were published about the prediction of preterm birth. Research on singleton pregnancy accounts for the majority, but there are some studies on twin pregnancy. Out of many factors, cervical length measured by ultrasound and fetal fibronectin test are suggested as a promising tool for purpose of the prediction of preterm birth. Some studies have commented that positive fetal fibronectin and short cervical length are associated with impending preterm birth in both symptomatic and asymptomatic twin pregnancies [15, 16]. The results of the current study also show that fetal fibronectin and cervical length are significant risk factors for preterm birth in symptomatic twin pregnancies. However, Conde-Agudelo et al. [17] showed in meta-analysis that the measurement of cervical length was of limited value to predict preterm birth in symptomatic twin pregnancy. The previous study by Fuchs F el al. study for accuracy of fetal fibronectin showed that the addition of cervical length ≤ 15mm on the results of fetal fibronectin test was lower positive but higher negative predictive value than fetal fibronectin only in prediction performance within delivery of 7 days [18]. In the current study, cervical length and positive fetal fibronectin were considered of critical co-factors, which is consistent with previous studies.

Among clinical factors during preterm labor, the patients’ subjective symptom (uterine contraction or labor pain) has not been evaluated by objective parameters in previous studies on preterm birth. However, labor is related to physiological changes of the myometrium and cervix which are divided into four phases of parturition [19, 20]. In phase 2 of parturition, the preparation of labor is associated with uterine activation and pregnant women perceive uterine contraction and sometimes complain of discomfort or pain. Although mild uterine activity may often be present before term, these contractions are usually painless with intrauterine pressure of less than 20 mmHg. In phase 3 of parturition, the uterine contraction intensity increases during labor, generating intrauterine pressures of 40mmHg on average (usually from 20 to 60 mmHg), resulting in patient’s painful symptom [21].

In the symptomatic uterine contractions or threatened preterm labor, patients may express pain characteristics (like cramping, firmness, squeezing) and intensity (as a self-reported pain score). Self-reported pain scores are widely used in pain assessment [10]. The usefulness of self-reported pain scores in term labor has been evaluated in the field of obstetrics and anesthesia [22]. In the current study, we tried to evaluate pain intensity using self-reported pain scores in preterm labor. As far as we know, this is the first study to evaluate the clinical usefulness of NRS pain scores in predicting the risk of preterm birth in symptomatic twin pregnancy.

As a result, we showed that NRS as pain intensity was critical factor in progression of preterm labor. Self-reported NRS pain score with known risk factors of cervical length and fetal fibronectin test improves the diagnostic accuracy of preterm delivery within 7 days up to 91.7 %. In the clinical management of patients with threatened preterm birth, the prediction of impending preterm birth within 2 or 7 days is the most important to optimize pregnancy and neonatal outcomes. Based on this prediction, the physicians must make decisions regarding several interventions, such as transfer to other hospitals and the use of corticosteroid, magnesium sulfate or tocolytics [23, 24].

Although we used cross-validation for indirect verification of our model, our study has some limitations. It was based on retrospective and single center data with a limited number of cases. As a result, several critical factors were significantly different between women with high NRS and those with low NRS (parity, frequency of uterine contractions, cervical length/dilatation, and tocolytics). In the study population, the use of tocolytics or steroid was at the discretion of attending physician. As a results, NRS was a significant risk factor for preterm birth even after adjustment for these factors. However, we could not split the study population into training and test set.

Larger studies in prospective and multicenter settings are needed to confirm the results of the current study. In addition, the findings of the current study should be further evaluated in other study populations such as in singleton pregnancy. Moreover, a higher NRS pain score may be related to other obstetric or gynecologic conditions [25]. Further studies regarding the NRS pain score in pregnant women with other obstetric or gynecologic conditions may be needed to respond to this case. The molecular mechanism regarding high NRS pain scores needs to be evaluated. The relationship between a high NRS pain score and other major markers such as intra-amniotic infection, cervicovaginal cytokines, and other vaginal or amniotic fluid markers should be evaluated in further studies [15, 26–28].

Lastly, we suggested a static nomogram for the prediction of preterm birth within 7 days in Fig. 3 and posted them online (http://lsy.io/nomogram/painscore) for easy access to promote clinical use. Further prospective studies are needed to verify the clinical usefulness of this suggested normogram.

## Conclusions

The accurate prediction of preterm birth will result in reduced neonatal complications by proper therapeutic strategies at the appropriate time. The developed nomogram will enable the accurate estimation of preterm birth by physicians. It might help twin pregnancies receive proper management of preterm labor. As a result, unnecessary hospitalization and the overuse of tocolytics or antibiotics will be reduced and neonatal outcomes will be improved.

## Data Availability

The datasets used and analyzed during the current study are available from the corresponding author on reasonable request.

## Abbreviations

PTB: Preterm birth
NRS pain score: Numerical rating scale pain score
AUC: Area under the receiver operating characteristic curve
ROC: Receiver operating characteristic curve
CL: Cervical length
FBN: Fetal fibronectin
